# Slowing of Event-Related Potentials in Primary Progressive Aphasia. A case report

**DOI:** 10.1100/tsw.2009.67

**Published:** 2009-07-14

**Authors:** Salvatore Giaquinto, Francesca Ranghi

**Affiliations:** IRCCS San Raffaele Rehabilitation Hospital, Rome, Italy

**Keywords:** primary progressive aphasia, dementia, event-related potentials

## Abstract

Primary Progressive Aphasia (PPA) is a rare and insidious language impairment that worsens over time. It belongs to the group of fronto–temporal dementias. This study was aimed at assessing the role of speed of cognitive abilities, such as word recognition, in PPA. The design is a single-case, longitudinal study. A male patient suffering from PPA was enrolled and 15 healthy older adults were the control group. An event-related electrical potential connected with word recognition, namely the N400, was delayed by 200 msec at baseline compared to healthy controls and progressively deteriorated. One year later, the delay was greater and two years later the potential had disappeared. Reduced speed of processing is an early pathological factor that negatively affecting higher cognitive functions in APP. Event–related electrical potentials are recommended in the field of aphasia and cognitive decline. They permit observation of a speed decline in higher cognitive abilities, when pathological changes at a central level begin and language comprehension seems to be unaffected.

## INTRODUCTION

A recent article by our group indicated that aging affects the various stages of perceptual processing and language comprehension in different ways[1]. Some correlates of lexical access were found to be stable with age in healthy elderly participants. The study also confirmed the possible applications of event-related potentials (ERPs) to cognitive decline. These responses can be recorded from the cortex by using verbal material on a millisecond-to-millisecond basis. Among the ERPs, the so-called N400 has possible functional relation to the lexical-semantic process. The N400 was identified in normal subjects presented with different seven-word sentences[2]. This ERP can be a useful model because it is a well-defined wave, permitting the measure of four parameters, namely latency, peak latency, amplitude, and duration, by using an oddball paradigm[3]. The role of the N400 in on-line semantic and lexical access accounts for its application to the field of dementia and amnestic syndromes[4,5]. More recently, in mild cognitive impairment, abnormalities of the P600 (a positive component recordable at a time interval longer than that of the N400) were associated with an increased risk of subsequent progression to dementia[6,7].

A question arises as to whether N400 recording can monitor the course of primary progressive aphasia (PPA), a rare neurological syndrome that impairs language capabilities and can help to predict the likelihood of Alzheimer's disease[8,9]. Naming objects, pronouncing words, verb tenses, and grammar can be severely affected. Reading can be difficult as well. PPA is insidious and worsens over time. The present report deals with a case of PPA followed for 2 years.

## METHODS

### Participant

A 70-year-old, right-handed male patient suffered over the last 2 years from a slowly progressing fluent aphasia, which apparently did not interfere with word comprehension. The onset was insidious and a gradual worsening of word finding and object naming took place. There were anomic aphasia with paraphasias and moderate impairment of speech repetition. Comprehension of spoken and written language was intact. With the exception of a mild constructional difficulty, other areas of cognition and behavior appeared preserved, and he continued to hold his job (gardening). The patient appeared smiling and happy to meet the hospital staff, but he was always unable to tell a single name. Diagnostic criteria were the intact premorbid language function, the absence of significant apathy, disinhibition, forgetfulness of recent events, visuospatial impairment, visual recognition deficit, or sensory-motor dysfunction in the initial 2 years of illness. General and neurological examination, blood pressure, and laboratory testing, including thyroid hormones, folate, and cyanocobalamin, were normal. There was no history of traumatic brain injury. The MRI showed mild cortical atrophy and the SPECT revealed reduced perfusion in both parietal and temporal areas of the left hemisphere.

Moreover, the following scales were applied: (1) CDR scale for dementia[10], (2) Mini-Mental State Examination[11], (3) Milan Overall Dementia Assessment[12], and (4) ENPA scale for evaluation of the Italian language[13]. At the first visit, the CDR score was 1 and Mini-Mental State 21. The patient was retested after 1 and 2 years. At the last examination, the CDR score was 2. ERPs were recorded at baseline, and 1 and 2 years later.

Fifteen age-matched volunteers comprised the control group; seven females and eight males. Italian was the first language of all participants. The mean age of the group was 65.1 years (S.D. 6.2). Their mean years of education was 12.5 years (S.D. 2.5). They were recruited from a senior club in the district. Their hearing was normal, and no neurological complaints or usage of drugs were reported.

The ethics committees and regulatory authorities approved the study protocol, and all participants gave their informed consent.

### Design

We conducted a prospective study of the patient with the last follow-up at 2 years. The N400 recording is a routine examination at our rehabilitation center. The method employed an oddball protocol where digitized words were presented in an auditory condition, and the target stimuli were represented by common names of animals (N = 20), i.e., by frequently encountered words in normal conversations[14]. Other common words were the nontarget stimuli (N = 80).

Subjects sat in comfortable, padded chairs in a sound-attenuated room with a fixation point. They had to press a button with their right thumb to signal that they had detected a target word. Reaction times to targets were recorded together with the number of false responses and omissions. Stimuli were delivered binaurally through earphones. The mean output level was 65 dB peSPL. Before the recording, three words were delivered through the earphones and subjects were invited to repeat them to check that the words were audible and correctly discriminated.

## Procedure

EEG signals were recorded from six sites using Ag/AgCl electrodes and referenced to the tip of the nose. Electrodes were placed according to standard International 10-20 System locations (F3, F4, C3, C4, P3, P4). One channel was allotted for ocular movement. Previous studies at our laboratory clearly showed an anterior location of brain waves related to word auditory processing, mainly at the frontal and central levels. Electrode impedance was always lower than 5 KΩ. Recording parameters were: 0.2–30 Hz bandpass, digitized at 256 Hz for 1 sec; interstimulus interval 2.3 sec. Traces contaminated by ocular artifacts greater than 80 μV were automatically rejected.

## Instrumentation

An EbNeuro machine was used. High-quality calibrated amplifiers were used and the data were stored on a hard disk. Traces were visually inspected off-line as a second control, with the possibility of manual rejection of contaminated traces. Averaging took place for responses to both target and nontarget stimuli. The words were digitally recorded by a professional female speaker and the audio files were edited to remove any silent period at the beginning of each sound. The mean duration of the words was 777 msec (S.D. 35).

## RESULTS

In this report, we compared ERPs obtained from a subject suffering from PPA with those from a group of healthy age-matched volunteers. The N400 in the control group had the following mean values: latency 308 msec (S.D. 58), duration 271 msec (S.D. 54), mean peak latency 441 msec(S.D. 84), and mean amplitude 3.6 μV (S.D. 1.6). The patient at baseline showed the following values: latency 510 msec, duration 400 msec, peak latency 610 msec, and amplitude 5.0 μV (Fig. 1).

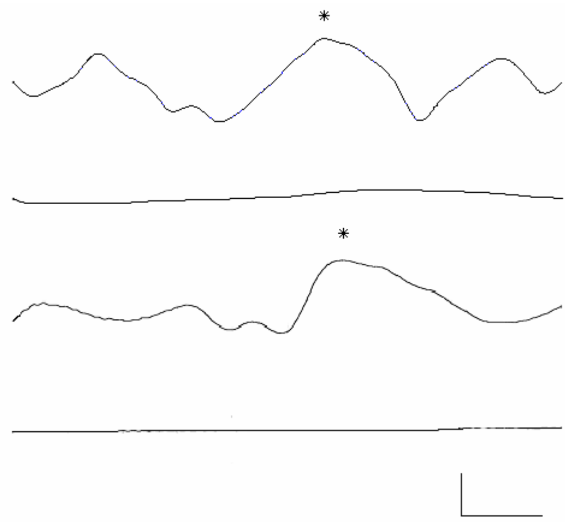

**FIGURE 1.** Averaged traces recorded from F3 (left frontal electrode). The N400 is marked by an asterisk (negative upward). Up: normal 80-year-old male subject. Down: a 70-year-old male patient at first examination, approximately 2 years after the beginning of language problems. The straight lines are the electro-oculograms. Time calibration: 200 msec. Amplitude calibration: 4 μV.

After 1 year, latency had increased, and amplitude and duration had decreased. After 2 years, the potential was no longer recognizable (Fig. 2).

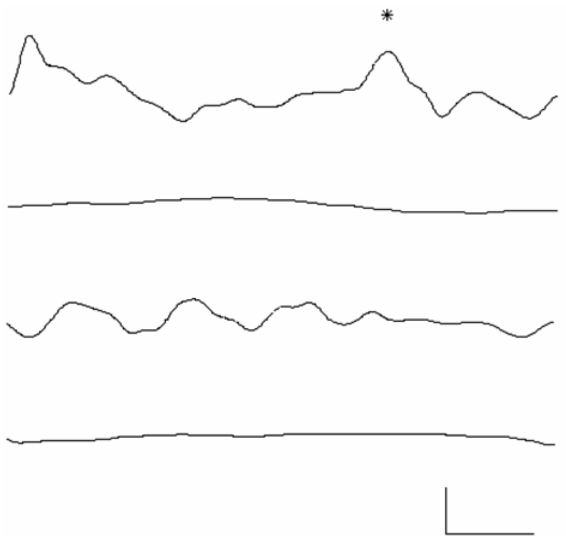

**FIGURE 2**. The same patient as in Fig. 1, 1 year (up) and 2 years later (down). Same indications as in the previous figure.

Mean choice reaction time (CRT) to target words was 893 msec (S.D. 78) in the control group. CRT was 1108 msec (S.D. 120) in the patient at baseline, and 1280 msec (S.D. 205) after 1 year. After 2 years from baseline, the task could not be executed. In the control group, the false alarms (FA) and the number of omissions (NO) were 2.63% of the total presentations. At baseline, the patient's performance had 20% of FA and NO, which became 52% after 2 years.

## DISCUSSION

At the end of the 19th century, the philosopher Galton stated that individuals having faster intellectual abilities also had better intelligence. This theory was short lived because it stemmed from associationistic psychology. It was argued that intelligence was independent of speed. Rather, complexity of reasoning was deemed much more important. However, by 1970, Computer Science had demonstrated that speed is associated with highly complex calculations. A faster computer not only saves time, but permits the solution of problems of higher complexity. A computer that has a 500-MHz clock is doing something 500 million times per second. A 1-GHz computer does something 1 billion times per second, a necessity in the field of artificial intelligence.

Unlike electronic hardware, impaired nervous tissue remains capable of processing information, although incompletely. Amyloid plaques and neurofibrillary tangles damage neuronal networks. The present observations indicate that slowing of information processing is the first step in cognitive decay. The N400 ERP has longer onset and an asymmetrical curve. In probability theory and statistics, "skewness" is a measure of the asymmetry of the probability distribution of a real-valued random variable. Several lines of research emphasize the concept of speed in neuropsychology (e.g., [15,16]). Direct age relations were found to the common factor as well as to first-order speed and memory factors. However, slowing is not always present in old age. Spectral analysis of EEG demonstrates the maintenance of the 9-Hz alpha rhythm[17] and the stability of N400 in normal aging[1].

N400 recording has a bearing in rehabilitation for the following reasons: (1) N400 is a diagnostic tool in the early phases of cognitive decline of the Alzheimer type, when language impairment begins; (2) N400 permits monitoring over time and therefore provides information on the time course of the disease; (3) the long and irregular nature of N400 suggests slow processing time in the brain and should encourage alerting relatives, caregivers, and speech therapists to speak slowly and simply, as the patient is unable to process the usual speech rate, i.e., 140 words per minute; (4) the absence of N400 implies that any verbal communication will fail; and (5) N400 is suitable for the study of drugs designed to affect cognitive performance.

In conclusion, neuropathological change in the nervous tissue hinders fast information processing. ERPs are useful tools for both diagnosis and monitoring of cognitive decline, to be used in parallel with neuropsychological examination. Slower information processing can be detected even in those cases with seemingly adequate word comprehension. The increase in latency and the wave asymmetry indicate a severe slowing of central processing. Later on, mechanisms are so slowed that temporal dispersion does not permit the cortical synchronization that builds up the peak of negativity during lexical access.

## ACKNOWLEDGMENTS

The research was supported by a grant from IRCCS San Raffaele Pisana, Rome, Italy. The authors declare that they have no conflict of interest. They are indebted to Mrs. Vissia Bachieca from RAI (Radio-Televisione Italiana) for the spoken words.
